# The Metropolitan Hospital Sunday Fund Meeting

**Published:** 1913-03-08

**Authors:** Lionel S. Lewis

**Affiliations:** St. Mark's Vicarage, Whitechapel, E.


*To the Editor of* The Hospital.

### The Metropolitan Hospital Sunday Fund Meeting.

*To the Editor of* The Hospital.

Sir,—Mr. Sydney Holland's accusation of want of candour against me made in your columns is most discreditable. Those who know me will smile, but for the benefit of those who do not I am entitled to ask for the insertion of this refutation in your paper.

Mr. Holland knows that I stated as one of the reasons for the falling off in the amount of our offertories to the Fund that we give one also for a hospital, for he immediately rose and asked the name of it, and at the request of the Lord Mayor I did not answer.

Unless the noise made by his party prevented his hearing my remarks, he must know that I stated that we gave more to that hospital than to the Fund, because at present we were dissatisfied with the Fund, for he could not have failed to have heard a clergyman with a stentorian voice rise and cry, "Shame!"

He continues his indelicate remarks a little further. My own subscription to the Anti-Vivisection Hospital is no secret. I mentioned it at a former meeting of the Fund in answer to a ridiculous charge of his of not caring for the sick poor. He sneeringly compares the 8s. 11d. sent to the Fund to the £1 1s. sent to the Anti-Vivisection Hospital. In addition to the above reasons stated in the room, the £1 1s. contained gifts from outsiders dissatisfied with the Fund. And the 8s. 11d. is a large sum for us, as our books will show. Since November 17, when a visitor put in 10s., no offertory has reached that sum. We have nothing to hide.

Other insults and misrepresentations I must endure, as I understand that your space is limited.—Your obedient servant,

Lionel S. Lewis.

St. Mark's Vicarage, Whitechapel, E., February 27.

See also note on page 612.

[This correspondence must now end.—Ed. The Hospital.]

Note.—Owing to pressure on our space we are obliged to hold over a long letter received from Mr. Walter Fearis with reference to his book, "The Treatment of Tuberculosis."

